# Energy and nutrient intakes in relation to National Nutrition Recommendations in a Norwegian population-based sample: the Tromsø Study 2015–16

**DOI:** 10.29219/fnr.v63.3616

**Published:** 2019-12-10

**Authors:** Marie W. Lundblad, Lene Frost Andersen, Bjarne K. Jacobsen, Monica Hauger Carlsen, Anette Hjartåker, Sameline Grimsgaard, Laila A. Hopstock

**Affiliations:** 1 Department of Community Medicine, UiT The Arctic University of Norway, Tromsø, Norway; 2 Department of Nutrition, Institute of Basic Medical Sciences, University of Oslo, Oslo, Norway; 3 Centre for Sami Health Research, Department of Community Medicine, UiT The Arctic University of Norway, Tromsø, Norway

**Keywords:** food frequency questionnaire, public health, population-based studies, adult, nutrient intake, energy intake

## Abstract

**Introduction:**

According to the Global Burden of Disease project, unhealthy diet accounts for most of the disease burden in Norway. Current recommendations on nutrient intake in Norway reflect those published in the evidence-based Nordic Nutrition Recommendations from 2012 (NNR2012).

**Aim:**

To study energy and nutrient intakes and compliance with the NNR2012 among women and men in a population-based study.

**Methods:**

A total of 15,146 participants (aged 40–99 years) completed a validated food frequency questionnaire (261 questions on food items, meals, and beverages) in the seventh survey of the Tromsø Study in 2015–16; 11,425 participants were eligible for the current analysis. Nutrient intake was estimated by a food and nutrient calculation system at the University of Oslo, Norway. We compared energy, macronutrient, and micronutrient intakes with the NNR2012.

**Results:**

In total, 85% of the women and men were not in compliance with the maximum recommended intake of saturated fat, and 40 and 77% of women and men, respectively, were not in compliance with the lowest recommended intake of fiber. More than 30% of women and 25% of men had a relatively high probability of inadequate intake of vitamin D, and more than 10% of the men had a relatively high probability of inadequate intake of vitamin B6 and vitamin C. More than 20% of women and men had a high probability of excessive intake of niacin, and almost 40% of women had a high probability of excessive intake of vitamin A.

**Conclusion:**

Although most participants were in compliance with NNR2012, a large proportion of participants had higher intakes than maximum recommended for saturated fat, and lower than recommended for fiber and vitamin D.

## Popular scientific summary

- Unhealthy diet accounts for most of the disease burden in Norway, and there is a need for updated population-based dietary data.
- We present energy and nutrient intakes in a large, Norwegian, population-based sample aged 40 years and above.
- Saturated fat intake was higher and fiber intake was lower than recommended by the Nordic Nutrition Recommendations from 2012.
- Data from the comprehensive Tromsø Study yield unique diet and health research possibilities.

Dietary habits are important predictors of health. Unhealthy diets contribute to the development of non-communicable diseases, such as cardiovascular disease, cancer, and diabetes (1). In 2016, the World Health Organization initiated the global strategy on diet, physical activity, and health, highlighting that populations’ nutrition recommendations should focus on ‘achieving energy balance and a healthy weight, limit energy intake from total fats and shift fat consumption away from saturated fats to unsaturated fats and towards the elimination of trans fatty acids, increase consumption of fruits and vegetables, and legumes, whole grains and nuts, limit the intake of free sugars and limit salt (sodium) consumption from all sources and ensure that salt is iodized’ (2). More than 100 countries (of which 30 in Europe) have provided national nutrition recommendations to enhance health, reduce the risk of nutrition-related diseases, and ensure adequate nutrient intake (3). These recommendations are designed to fit the specific food habits and access to foods in each population, and thus vary across countries. Nevertheless, in general, the recommendations all include a reduction in the intake of saturated fat, trans fat, salt, and added sugar (4).

The Norwegian Directorate of Health has created both food-based dietary guidelines and specific recommendations for nutrient intake. The food-based dietary guidelines consist of 12 recommendations specifying healthy food choices (e.g. whole grain products and lean meats) and which foods to reduce (e.g. processed meat, red meat, and salt) (5, 6). In Norway, current recommended nutrient intakes are based on the Nordic Nutrition Recommendations from 2012 (NNR2012). These specific nutrient recommendations have not been distributed to the general population as frequently as the more general, food-based dietary guidelines (7). In the NNR2012, nutrient recommendations are given for energy-providing nutrients as percentages of total energy intake (E%) from fats, carbohydrates, proteins, and alcohol; or as total amount (μg, mg, or g) of fiber, vitamins, and minerals per day (7).

During the last 25 years, three national nutrition surveys mapping food and nutrient intake have been conducted in the Norwegian adult population: Norkost 1 1994–95 (*n* = 3144 aged 16–79 years) (8), Norkost 2 1997 (*n* = 2672 aged 16–79 years) (9), and Norkost 3 2010–11 (*n* = 1787 aged 18–70 years) (10). Food frequency questionnaires (FFQs) were used in Norkost 1 and 2 (8, 9), and two repeated 24-hour dietary recalls and a food propensity questionnaire were used in Norkost 3 (10). In Norkost 3, the intake of saturated fat was higher and intake of carbohydrates, vitamin D, and folate were lower than recommended in both women and men. Surveillance of dietary intake in populations is important to address potential risks and to develop targets for dietary improvements (4, 11, 12). At present, there is a need for a current nutrient intake status in a Norwegian population-based sample. Therefore, the aim of this study was to provide an updated overview of energy and nutrient intakes in adult and elderly women and men and to identify the proportion that is not in compliance with the NNR2012, using data from a large Norwegian population-based study conducted in 2015–16.

## Methods

### The Tromsø Study

The Tromsø population-based study was conducted in the Tromsø municipality, located in Northern Norway. The Tromsø municipality consists of both urban and rural living areas and is similar to the general Norwegian population according to age (13), gender (13), and education level (14). The Tromsø Study consists of seven repeated surveys conducted between 1974 and 2016 (Tromsø 1–Tromsø 7), to which total birth cohorts and random samples were invited (15, 16). Thus, at each survey, previous and new participants are invited. The data collection process consisted of questionnaires and interviews, clinical examinations, and collection of biological samples, with response rates ranging from 65% to 79%. The present analyses are based on data from Tromsø 7 (2015–2016).

### Sample

**Fig. 1:**
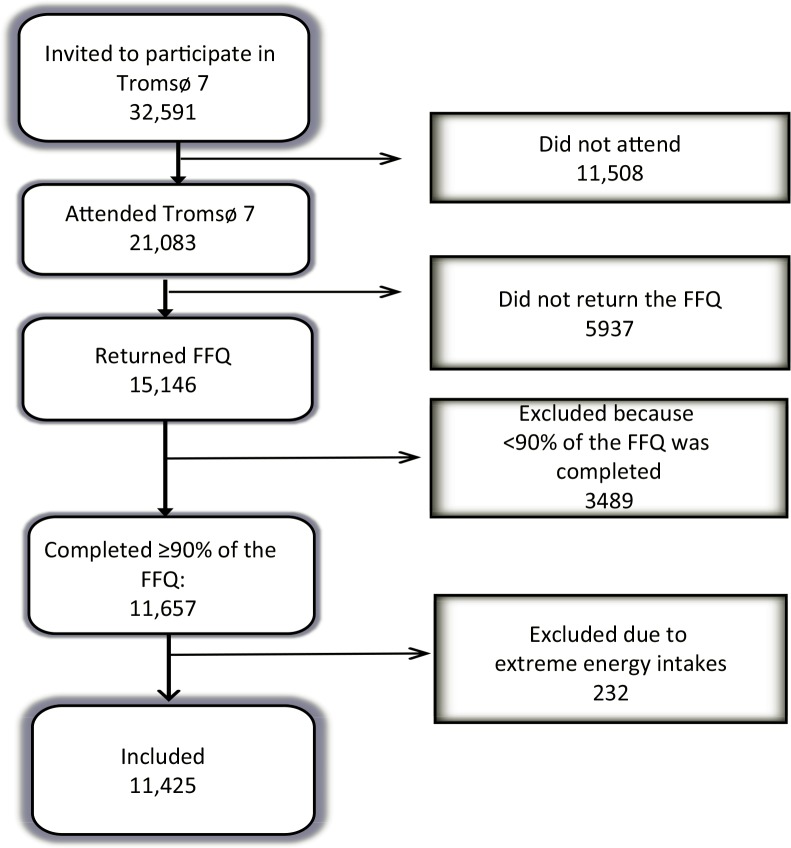
Flow chart of study sample. FFQ: food frequency questionnaire.

In Tromsø 7, all inhabitants of Tromsø municipality aged 40 and older were invited by mail (*n* = 32,591), and a total of 21,083 women and men aged 40–99 years attended (response rate 65%). After signing an informed consent form, participants were invited to complete several questionnaires, including a 13-page FFQ; attend clinical examinations; and have biological samples collected. A total of 15,146 participants completed the FFQ (response rate 72% of those participating in Tromsø 7 and 46% of those originally invited). Of these, we excluded participants who completed less than 90% of the frequency questions (*n* = 3489). Furthermore, to exclude highly unrealistic energy intakes, we excluded the 1% with the highest (above 21,267 kJ/day) and the 1% with the lowest (below 3,948 kJ/day) total energy intake (*n* = 232). Thus, a total of 11,425 participants (aged 40–96 years, 53% women) were included in our analyses, that is, 54% of the total number of participants in Tromsø 7 and 75% of the original FFQ sample (Fig. 1). Tromsø 7 is approved by the Regional Committee for Medical Research Ethics (REC North ref. 2014/940).

### Food frequency questionnaire

The FFQ was developed at the University of Oslo (UiO) to measure usual food intake in a Norwegian population and included questions about average intake of 261 different types of food, dishes, dietary supplements, meals, and beverages, including alcoholic beverages, during the last year. Validation studies have shown that this FFQ is suitable for estimating average energy and nutrient intake, as detailed elsewhere (17–19). The FFQs were handed out to participants when they attended the Tromsø 7 examination site. Study technicians gave the following short oral motivation as to why the participants should complete the FFQ: ‘this can give us important answers regarding dietary habits in the population, and reveal potential associations with lifestyle diseases, like diabetes’. The FFQ could be completed either on-site or at home and returned by mail. The front page of the FFQ included information on why data on food intake are important, and the first page contained explanations of how to complete the questionnaire. Data collection for Tromsø 7 started in March 2015 and was completed by the end of October 2016; the last FFQ was received in February 2017. All paper FFQs were manually checked by trained technicians, and necessary changes were made according to standard UiO criteria before they were scanned for digital storage (Supplementary Fig. 1). After scanning, raw data were checked according to metadata criteria (date format, number of decimals, etc.) before they were sent to the UiO for computation of food and nutrient intakes. Energy and nutrient intakes were calculated using the food database KBS AE14 and KBS software system at the UiO (KBS, version 7.3.). The food database KBS AE14 is based on the Norwegian food composition tables from 2014 to 2015 (20), supplemented with data from calculated recipes and other databases. Intake from both food and dietary supplements were included in the nutrient calculations. Thereafter, dietary data were imported into EUTRO at UiT the Arctic University of Norway (Supplementary Fig. 1). EUTRO is an IT-solution that gathers all data from the Tromsø Study into one database (21).

### Information about demographics and lifestyle factors

To compare characteristics between attenders and nonattenders in Tromsø 7, we included data on age and sex from the Norwegian Population Registry. Self-reported data on current smoking (yes/no), education level (primary, upper secondary, tertiary short [<4 years], and tertiary long [≥4 years]), leisure-time physical activity level based on the Saltin and Grimby questionnaire (22) (sedentary, light, and moderate/vigorous), and subjective memory complaints (yes/no) were available from Tromsø 7 questionnaires.

### Statistical analyses

We performed descriptive analyses of age (years), smoking, education level, and leisure-time physical activity level for women and men in 10-year age groups (Table 1). Only 11 participants (0.1%) were older than 90 years and were included in the 80+ age group. We performed sex-specific, descriptive analyses to present median energy, macronutrient intake, and micronutrient intake, as well as the proportion of participants that were not in compliance with the NNR2012 (Tables 2–4). The recommendations from NNR2012 vary in the form they are given. In some cases, the recommended intakes are given as an interval to be reached (e.g. protein, carbohydrate, and total fat). For these, we present both total proportion not in compliance (below or above recommended interval combined) and the proportion below the recommended interval and the proportion above recommended separately (Table 2). In other cases, recommendations are given as a ‘minimum to reach’ (e.g. fiber) or as a ‘maximum to not exceed’ (e.g. saturated fat, and alcohol). For these the proportion not in compliance is presented as those below ‘minimum to reach’ or as above ‘maximum to not exceed’ (Table 2). We used Student’s *t*-test (for continuous variables) and chi-square tests (for categorical variables) to investigate differences between attenders and nonattenders, and between those excluded and included in the final analyses (Supplementary Tables 1 and 2, respectively). We also performed descriptive analyses to present sex- and age-specific (10-year age groups: 40–49, 50–59, 60–69, 70–79, and 80+) mean energy intake, macronutrient intake, and micronutrient intake with 95% confidence intervals (Supplementary Tables 3–6). The units of measure presented for each macronutrient and micronutrient are in accordance with the NNR2012 (7) and the Norwegian Directorate of Health (23); intake of energy-providing nutrients (except for fiber) are presented as E%; and fiber intake, macronutrient intake, and micronutrient intake are presented as absolute intake per day. Micronutrient intake is also presented as intake per 10 MJ, to account for total energy intake and to investigate the quality of the diet.

**Table 1:** Characteristics of the study population by sex and 10-year age groups. The Tromsø Study 2015–16

	% N	Age (years)	Current smoking		Education level				Leisure-time physical activity level		
			Yes	No	Primary	Upper secondary	Tertiary education, short (<4 years)	Tertiary education, long (≥4 years)	Sedentary	Light	Moderate and vigorous
Total	11,425	57.5 (10.8)	12.6 (1,429)	87.4 (9,919)	20.6 (2,327)	27.1 (3,064)	20.5 (2,321)	31.8 (3,590)	13.0 (1,455)	58.8 (6,557)	28.2 (3,149)
Women	53.4 (6,104)	56.9 (10.7)	13.5 (820)	86.5 (5,240)	21.0 (1,270)	25.5 (1,539)	18.4 (1,112)	35.1 (2,122)	12.7 (751)	65.0 (3,857)	22.4 (1,327)
40–49	30.0 (1,833)	44.7 (2.84)	12.3 (224)	87.7 (1,603)	6.02 (110)	22.7 (415)	21.4 (392)	49.8 (911)	13.4 (244)	57.1 (1,040)	29.5 (538)
50–59	29.7 (1,813)	54.5 (2.93)	16.3 (294)	83.7 (1,507)	14.7 (264)	28.0 (504)	20.0 (359)	37.3 (671)	11.1 (198)	66.6 (1,194)	22.3 (400)
60–69	27.0 (1,646)	64.2 (2.89)	14.2 (231)	85.8 (1,400)	30.5 (497)	26.9 (438)	16.0 (260)	26.6 (433)	11.5 (184)	71.8 (1,145)	16.7 (266)
70–79	11.1 (679)	73.4 (2.70)	9.40 (63)	90.6 (607)	48.5 (320)	23.5 (155)	13.5 (89)	14.6 (96)	15.0 (92)	67.2 (412)	17.8 (109)
80+	2.2 (133)	83.1 (3.22)	6.11 (8)	93.9 (123)	61.2 (79)	20.9 (27)	9.3 (12)	8.5 (11)	29.2 (33)	58.4 (66)	12.4 (14)
Men	46.6 (5,321)	58.1 (11.0)	11.5 (609)	88.5 (4,679)	20.1 (1,057)	29.0 (1,525)	23.0 (1,209)	27.9 (1,468)	13.5 (704)	51.7 (2,700)	34.9 (1,822)
40–49	26.9 (1,433)	44.8 (2.81)	11.6 (165)	88.4 (1,261)	10.2 (145)	29.9 (427)	22.6 (323)	37.3 (533)	14.6 (208)	42.0 (597)	43.4 (618)
50–59	27.5 (1,464)	54.4 (2.85)	13.1 (191)	86.9 (1,264)	17.8 (259)	29.4 (428)	25.8 (375)	27.1 (394)	12.2 (176)	52.6 (762)	35.3 (511)
60–69	28.6 (1,521)	64.3 (2.85)	12.4 (188)	87.6 (1,326)	25.7 (386)	27.4 (412)	22.4 (336)	24.6 (369)	13.7 (204)	57.2 (855)	29.1 (435)
70–79	14.4 (768)	73.4 (2.69)	7.24 (55)	92.8 (705)	29.7 (221)	29.5 (220)	19.7 (147)	21.1 (157)	12.6 (93)	56.9 (419)	30.5 (225)
80+	2.54 (135)	82.9 (2.81)	7.52 (10)	92.5 (123)	36.2 (46)	29.9 (38)	22.1 (28)	11.8 (15)	18.7 (23)	54.5 (67)	26.8 (33)

Results are given as mean (standard deviation) or as percentage (number).

**Table 2:** Energy and macronutrient intake in women and men, and the percentage of participants not in compliance with the NNR2012. The Tromsø Study 2015–16

Macronutrients	NNR2012 recommendation	Median	25^th^ – 75^th^ percentile	Not in compliance (%)
**Women**				
Energy (MJ/day)		8.5	7.0–10.4	
Protein (E%)	10–20	18	16–19	18 (0.1 below, 17.4 above NNR2012)
Fat (E%)	25–40	35	31–38	19 (3.2 below, 16.2 above NNR2012)
-Saturated fat (E%)	<10	12	11–14	85
-Trans fat (E%)	<1	0.3	0.2–0.4	0
-Monounsaturated fat (E%)	10–20	13	11–15	14 (11.8 below, 1.8 above NNR2012)
-Polyunsaturated fat (E%)	5–10	6	5–7	27 (25.2 below, 1.7 above NNR2012)
-Omega 3 (E%)	≥ 1	1.4	1.1–1.8	18
Carbohydrates (E%)	45–60	42	38–46	69 (68.6 below, 0.3 above NNR2012)
-Fiber (g/day)	>25 g/day	27	22–34	40
-Added sugar (E%)	<10	5	3–7	7
Alcohol (E%)	<5	2	0.5–4.1	20
**Men**				
Energy (MJ/day)		10.3	8.4–12.5	
Protein (E%)	10–20	17	16–19	14 (0.2 below, 13.8 above NNR2012)
Fat (E%)	25–40	34	31–38	17 (4.7 below, 12.7 above NNR2012)
-Saturated fat (E%)	<10	12	11–14	84
-Trans fat (E%)	<1	0.3	0.2–0.4	0.1
-Monounsaturated fat (E%)	10–20	12	11–14	15 (13.6 below, 1.2 above NNR2012)
-Polyunsaturated fat (E%)	5–10	6	5–7	27 (24.9 below, 2.0 above NNR2012)
-Omega 3 (E%)	≥ 1	1.4	1.1–1.8	16
Carbohydrates (E%)	45–60	43	39–46	67 (66.4 below, 0.4 above NNR2012)
-Fiber (g/day)	>35 g/day	27	22–34	77
-Added sugar (E%)	<10	5	3.3–7.2	10
Alcohol (E%)	<5	3	1.0–5.4	28

**Table 3:** Micronutrient intake in women, and the percentage of women with micronutrient intake below or above specified NNR2012 values. The Tromsø Study 2015–16

Micronutrients	NNR2012 recommendation						Percentage of participants			
	LI	AR	RI	UL	Median	25^th^ – 75^th^ percentile	Below LI	Below AR	Above RI	Above UL
Vitamin A (RE/day)	400	500	700	1,500^1^	1,280	915–1,753	1	3	88	38
Vitamin D (μg/day)	2.5	7.5	10	100	10.6	6.4–19.0	2	33	53	0
Vitamin E (α-TE/day)	3	5	8	300	18.1	12.7–27.0	0	0	95	0
Vitamin B6 (mg/day)	0.8	1.0	1.2	25	1.91	1.4–2.8	1	5	87	0
Vitamin B12 (μg/day)	1	1.4	2	*	7.2	5.5–9.5	0	0	100	*
Vitamin C (mg/day)	10	50	75	*	151	103–213	0	4	88	*
Thiamin (mg/day)	0.5	0.9	1.1	*	1.86	1.39–2.6	0	3	90	*
Riboflavin (mg/day)	0.8	1.1	1.3	*	2.55	1.9–3.6	1	4	93	*
Niacin (NE/day)	9	12	15	35	24	18.3–32.8	1	3	88	21
Folate (μg/day)	100	200	300^2^	*	334	256–435	0	9	60	*
Calcium (mg/day)	400	500	800	2,500	1,001	737–1,335	3	7	69	2
Phosphorus (mg/day)	300	450	600	3,000	1,719	1,376–2,128	0	0	100	5
Potassium (g/day)	1.6	*	3.1	*	4.59	3.7–56.4	0	*	90	*
Magnesium (mg/day)	*	*	280	*	390	315–482	*	*	85	*
Iron (mg/day)	5^3^	6^3^	9^3^	60	10.5	8.4–13.8	2	6	67	1
Zinc (mg/day)	4	5	7	*	11.9	9.4–16	0	1	93	*
Copper (mg/day)	0.4	0.7	0.9	5.0	1.25	0.97–1.7	0	6	81	1
Selenium (μg/day)	20	30	50	300	57	44–77	0	4	62	0
Iodine (μg/day)	70	100	150	600	288	210–386	0	2	91	4

1. Postmenopausal cut of level used: 1,500 μg/day.

2. Postmenopausal cut off level used: 300 μg/day.

3. Postmenopausal recommendations for iron are applied.

*Not established.

*NNR2012: Nordic Nutrition Recommendations 2012; LI: lower intake level; AR: average requirement; RI: recommended intake; UL: upper intake level.

**Table 4:** Micronutrient intake in men, and the percentage of men with micronutrient intake below or above specified NNR2012 values. The Tromsø Study 2015–16

Nutrients	NNR2012 recommendation						Percentage of participants			
	LI	AR	RI	UL	Median	25th – 75th percentile	Below LI	Below AR	Above RI	Above UL
Vitamin A (RE/day)	500	600	900	3,000^1^	1,324	945–1,801	4	7	78	4
Vitamin D (μg/day)	2.5	7.5	10	100	11.4	7.5–19.3	1	25	58	0
Vitamin E ( α-TE/day)	4	6	10	300	18.6	13.2–27.5	0	1	90	0
Vitamin B6 (mg/day)	1.0	1.3	1.5	25	1.96	1.5–2.6	3	12	77	0
Vitamin B12 (μg/day)	1	1.4	2	*	8.8	6.6–11.4	0	0	100	*
Vitamin C (mg/day)	10	60	75	*	122	82–177	0	12	79	*
Thiamin (mg/day)	0.6	1.2	1.4	*	1.93	1.5–2.5	0	8	83	*
Riboflavin (mg/day)	0.8	1.4	1.7	*	2.77	2.1–3.6	0	6	87	*
Niacin (NE/day)	12	15	18	35	26.5	21–33.8	2	6	86	22
Folate (μg/day)	100	200	300	*	332	260–420	0	9	61	*
Calcium (mg/day)	400	500	800	2,500	1,147	840–1,517	2	5	78	2
Phosphorus (mg/day)	300	450	600	3,000	1,977	1,589–2,433	0	0	100	9
Potassium (g/day)	1.6	*	3.5	*	5.13	4.2–6.3	0	*	89	*
Magnesium (mg/day)	*	*	350	*	435	353–538	*	*	76	*
Iron (mg/day)	7	7	9	60	11.3	9–14.4	9	9	74	0
Zinc (mg/day)	5	6	9	*	13.6	10.8–17.2	0	1	88	*
Copper (mg/day)	0.4	0.7	0.9	5.0	1.27	1.0–1.7	0	5	84	1
Selenium (μg/day)	20	35	60	300	68	52–87	0	4	62	0
Iodine (μg/day)	70	100	150	600	337	249–446	0	1	95	7

1. Vitamin A UL: 3,000.

*Not established.

*NNR2012: Nordic Nutrition Recommendations 2012; LI: lower intake level; AR: average requirement; RI: recommended intake; UL: upper intake level.

In addition to presenting median (Table 2) and mean (Supplementary Table 3) energy intakes, we investigated underreporting of energy intake by using the Goldberg method (24). We used a physical activity level ≤1.2 (chair-bound or bed-bound) to compute the proportion of participants that most likely underreported their energy intake as described by Black et al. (24).

Macronutrient intake is presented as medians with 25–75 percentiles in addition to percentage of participants not in compliance with the NNR2012. Micronutrient intake is presented as at risk for inadequate intake or excessive intake as recommended by the NNR2012. These were defined as: lower intake level (LI) – ‘values below LI could lead to clinical deficiency symptoms in most individuals’ (7, p. 50); average requirement (AR) – ‘the lowest long-term intake level of a nutrient that will maintain a defined level of nutritional status in an individual’ (7, p. 46); recommended intake (RI) – ‘the amount of a nutrient that meets the known requirement and maintains good nutritional status among practically all healthy individuals in a particular life stage or gender group’ (7, p. 48); and upper intake level (UL) – ‘the maximum level of long-term daily nutrient intake that is unlikely to pose a risk of adverse health effects in humans’ (7, p. 49). As recommended by the NNR2012, we present the proportion of women and men that had very high probability of inadequate intake (intakes below LI), relatively high probability of inadequate intake (intakes below AR), minimal probability of inadequate intake (intakes above RI), and a high probability of excessive intake (intakes above UL) of micronutrients (7, p. 71). However, not all micronutrients have established cut-off values, especially for UL. So excessive intakes could not always be estimated. We used STATA 15 (STATA Corp LP, College Station, Texas, USA) to perform all analyses. P-values were considered significant at a 0.05 level.

## Results

Among the 11,425 participants included in the analyses, 53% were women. The mean age was 57 and 58 years among women and men, respectively. A total of 13% of the participants were current smokers. Education level was inversely associated with age (*P* < 0.01). More than half of the participants reported light leisure-time physical activity level (Table 1). Compared to those not returning the FFQ, the final sample included a higher proportion of women, non-smokers, and participants with higher education (Supplementary Table 2).

### Energy intake

Women and men had a median energy intake of 8.5 and 10.3 MJ/day, respectively (*P* < 0.001) (Table 2). The mean energy intake was inversely associated with age (*P* < 0.001) (Supplementary Table 3). When adjusted for age, the mean energy intake was positively related to leisure-time physical activity level (*P* < 0.001) (results not shown in the tables). Current smokers had a higher energy intake than that of non-smokers (*P* = 0.003) (results not shown in the tables). Based on the Goldberg method, approximately 20% of the study population likely underreported their energy intake.

### Macronutrient intake

More than 80% of both women and men were in compliance with the recommended intakes of protein, total fat, trans fat, monounsaturated fat, omega-3 fatty acids, and added sugar in the NNR2012 (Table 2). A total of 95% of the women and 97% of the men had trans fat intakes below 0.5 E% (results not shown in tables). About 85% of both women and men had intakes that were higher than the maximum NNR2012 recommendations of saturated fat, and about 25% of women and men had intakes that were lower than the minimum NNR2012 recommendations of polyunsaturated fat (Table 2). Almost 70% of both women and men were not in compliance with the intake of carbohydrates recommended by the NNR2012, and 40 and 77% of women and men, respectively, were not in compliance with the lowest recommended intake of fiber. Moreover, 20 and 28% of women and men, respectively, had intakes above the maximum level of alcohol recommended by the NNR2012 (Table 2).

### Micronutrient intake

Almost none of the participants had very high probability of inadequate intake (below LI) of any of the micronutrients examined (Tables 3 and 4). The exception was 4 and 9% of men, who, according to our data, had a very high probability of inadequate intake of vitamin A and iron, respectively (Table 4). However, a larger proportion of the population had a relatively high probability of inadequate intake (below AR) of some nutrients (Tables 3 and 4).

In women, 33% had a relatively high probability of inadequate intake of vitamin D (Table 3). In addition, 6–9% of women had a relatively high probability of inadequate intake of iron, copper, calcium, and folate (Table 3). For all other micronutrients, less than 6% of women had a relatively high probability of inadequate intake. More than 50% of women had a minimal probability of inadequate intake of vitamin D, folate, calcium, iron, and selenium. For the other micronutrients, more than 80% of women had a minimal probability of inadequate intake. A total of 38 and 21% of women had a high probability of excessive intake of vitamin A and niacin, respectively.

In men, 25% had a relatively high probability of inadequate intake of vitamin D (below AR) (Table 4). Twelve percent had a relatively high probability of inadequate intake of both vitamin B6 and vitamin C. About 6–9% of men had a relatively high probability of inadequate intake of riboflavin, niacin, vitamin A, thiamin, folate, and iron (Table 4). For all other micronutrients, less than 6% of men had a relatively high probability of inadequate intake. For vitamin D, 58% of the men had minimal probability of inadequate intake (above RI). For vitamin A, vitamin B6, vitamin C, folate, calcium, magnesium, iron, and selenium about 60–80% of men had a minimal probability of inadequate intake. For all other micronutrients, more than 80% of men had a minimal probability of inadequate intake. A total of 22 and 9% of men had a high probability of excessive intake of niacin and phosphorus, respectively.

## Discussion

### Energy intake

Energy intake requirements depend on the body size and energy expenditure. The median and mean energy intakes in our population correspond to the approximate reference energy requirements for women and men with a sedentary to average activity level (7, 23). Most of our study sample reported a light physical activity level, and the reference value for energy intake for women and men with a sedentary to average activity level seems to be a good comparison. Although the data collection methods and sample age range differed, the mean energy intake in our population is similar to what was observed in Norkost 3 in 2011 using two 24-h dietary recalls in 18–70-year-olds (10). In our study, we estimated that 20% of the participants most likely underreported their food intake. This is comparable to what has previously been reported from other population-based surveys using FFQs (10, 17, 25). Underreporting will affect observed macronutrient and micronutrient intake to various extents, in that the observed intake is lower than the actual intake. However, we cannot know which of the nutrients are affected most by possible underreporting. Also, underreporting was investigated after the exclusion criteria specified earlier, meaning that the proportion of underreporters would have been even higher if we did not exclude those who submitted incomplete questionnaires. We did however investigate underreporting in what we considered as a reliable sample.

### Macronutrient intake

A large proportion of women and men were not in compliance with the intakes of carbohydrates and fiber recommended by the NNR2012, and had intakes higher than the maximum recommended intake of saturated fat. Although within recommended interval, intakes of total fat (E%) and protein (E%) were relatively high compared to the NNR2012 (25–40% and 10–20 E%, respectively). Thus, the slightly below recommended intake of carbohydrates (E%) should be of little concern because it basically reflects the relatively high (although within recommended) total fat and protein intake. In addition, a substantial proportion of our study sample was not in compliance with recommended intakes of polyunsaturated fat and reported excessive intakes of alcohol.

The obesity epidemic, dieting, and weight-loss programs receive much attention in the media, and various sources have frequently advocated diets such as low-carb-high-fat diets. Such diets are characterized by a high saturated fat and low carbohydrate intake, as is reflected in our study sample (26). The possible population impacts of low-carb-high-fat diets have been discussed in a Swedish study (27). Deviations from recommended intakes of nutrients are cause for concern if they, at some point, could cause negative consequences on health due to low or high intakes. Examples of such macronutrients are added sugar, fiber, and saturated fat. The large majority of both women and men in our sample had less than 10 E% from added sugar. This corresponds to what was observed in Norkost 3, in which both women and men had a mean intake of 7.3 E% from added sugar (10). Intake of added sugar is reported to increase the risk for caries, overweight, and obesity (6), while high intake of fiber reduces the risk of colon cancer, esophageal cancer, cardiovascular diseases, type 2 diabetes, overweight, and obesity (6). The low intake of fiber found in our study is in accordance with other studies from Nordic countries (28, 29) and with results from national dietary surveys in 21 European countries (30).

Although most of our study sample was in compliance with the NNR2012 with regard to intake of total fat, more than 80% of women and men had higher intakes than the maximum recommended for saturated fat. Intake of saturated fat is reported to increase the risk for cardiovascular disease and overweight, but the discussion regarding the effect of saturated fat (alone) on health is ongoing (6). Our results are similar to those from other studies in Norway and Nordic countries (10, 28, 29), and from other European countries (30). However, it should be mentioned that most studies included in the review from Rippin et al. (30) used 24-hour recall interviews or dietary intake diaries, and are therefore not directly comparable to our results, as we used FFQs.

It is generally recommended that the intake of trans fat should be as low as possible and not higher than 1 E%. Almost all participants in our study had trans fat intakes below 1 E%. A total of 27% of our population was not in compliance with the recommended intake of polyunsaturated fat, as has also been found in several other studies from the Nordic countries (29–31). A substantial proportion of our population had alcohol intakes that were higher than the maximum recommended level. This is in accordance with Jonsdottir et al. (29), where 24% of women and 35% of men in the Nordic countries had higher intakes of alcohol than the recommended maximum.

### Micronutrient intake

Almost all participants complied with the recommended micronutrient intakes, with the exception of vitamin D and niacin, for which 33% of women had a relatively high probability of inadequate intake of vitamin D, and >20% of women had a high probability of excessive intake of niacin. In men, >20% had a relatively high probability of inadequate intake of vitamin D and >20% had a high probability of excessive intake of niacin.

Compared to the findings in Norkost 3 (10), our population had a relatively higher mean intake of vitamin D. This could be attributed to a high intake of fatty fish (like salmon), vitamin D fortification in foods, or vitamin D supplements. Indeed, nearly 40% of our sample reported that they took vitamin D supplements sometimes or daily (The Tromsø Study, unpublished material). Although the mean intake of vitamin D in our population was higher than that found in Norkost 3, a large proportion of women and men were still not in compliance with vitamin D recommendations. Intakes of vitamin D below recommended levels have been documented in several other studies (29, 31, 32). However, Rippin et al. (30) found that the intake of vitamin D was higher in Nordic countries compared to other European countries. They suggested that this could be due to fortification of foods or to the fact that people are aware that the limited access to sunlight in northern countries makes dietary vitamin D intake more important (30). In Norway, products like margarine and butter (10 μg/100 g), semi-skimmed milk (0.4 μg/100 g), and lactose-reduced milk (0.4 μg/100 g) (33) are fortified with vitamin D. A systematic review investigating micronutrient intakes and potential inadequacies among community-dwelling older adults from Western countries found that almost all participants had intakes of vitamin D that were lower than recommended (34). There was, however, a large range of vitamin D intake in our sample (from 0.33 to 80 μg/day), which may explain why a substantial proportion of women and men were at risk for inadequacy even though mean intake was quite high.

More than 20% of both women and men had a high probability of excessive intake of niacin. Health issues related to excess intake may include flushing (burning and itching of the face, arms, and chest) and stomach irritation (35). High intakes of niacin from dietary sources are not regarded as a health risk, and the recommended upper limit therefore mainly regards dietary supplements (35). The nutrient intake in our study is a combination of intake from dietary sources and dietary supplements. Most of the participants reported that they rarely or never took multivitamins or vitamin B supplements (75%). When we restricted the analysis to participants who seldom/never took supplements, 5 and 14% of women and men, respectively, had a high probability of excessive intake of niacin (results not shown in tables).

For some nutrients, the recommendations differ for pre- and postmenopausal women. The NNR2012 recommends an iron intake of 15 mg/day for premenopausal women and 9 mg/day for postmenopausal women, and the AR is 10 mg/day and 6 mg/day for pre- and postmenopausal women, respectively. The mean intake of iron was higher in women aged 50 years or younger compared to older women (13.7 μg/day and 11.8 μg/day, respectively). It is reasonable, based on the age of the women in our sample (all aged 40 years and above), to assume that the majority of women were postmenopausal; therefore, we applied the recommended intake for postmenopausal women in our analyses. Recommendations for the intake of vitamin A also differ for pre- and postmenopausal women (3000 RE/day and 1500 RE/day, respectively). We also chose to apply recommendations for postmenopausal women for vitamin A. Only 4% of the women had a high probability of excessive intake of vitamin A when applying the upper recommended level for premenopausal women, while almost 40% had a high probability of excessive intake when using the upper recommended level for postmenopausal women. Hypervitaminosis A (both acute and chronic) includes a wide range of symptoms that can affect the nervous system, the circulatory system, the musculoskeletal system, and internal organs, among others (36). Blomhoff et al. (36) showed that vitamin A intake in Nordic countries is higher than that in other countries, but that health issues due to excess intakes rarely occur.

### Strengths and limitations

The major limitation when using an FFQ is that data are self-reported, which could lead to misreporting, either consciously or unconsciously. As mentioned previously, 20% of our sample population likely underreported their dietary intakes, which means that most nutrient intakes were likely higher than presented here. However, this study also has several strengths. The FFQ distributed to the participants is very comprehensive and contains information on 261 foods, meals, dietary supplements, and beverages (including alcoholic beverages). This FFQ has been validated (17–19) and is, in a study of this size, the obvious tool of choice to assess usual food and nutrient intake in a Norwegian population. Another important strength is the large sample of 11,425 people from a population-based survey. Moreover, the overall participation in Tromsø 7 was high (65%), and after exclusion (by the criteria specified above), 35% of the total population in Tromsø aged 40 years and above were included in final analyses. It would, however, have been even more preferable if a larger age range of the adult population had been included, and that our study is restricted to a population aged 40 years and older could therefore also be regarded as a limitation.

Nonattenders tend to differ from attenders in population-based studies such as the Tromsø Study (15, 37–39). Although the differences between those included and excluded in the final sample are statistically significant for several variables (e.g. age, education level, and subjective memory complaints), the differences in the mean values were relatively minor. We, therefore, believe that the final study sample does not differ much from the non-attenders in Tromsø 7 (in relation to age and sex) or from those who attended Tromsø 7 (but were not included in the final sample). The Tromsø municipality includes both urban and rural populations and is believed to be comparable to most other populations in the same age range in Norway. It is, therefore, reasonable to assume that the external validity in our study is relatively high; the results reflect the dietary habits in Tromsø and are likely to reflect the situation of other Norwegian adults aged 40 years and older.

## Conclusion

In this adult Norwegian population-based sample, most participants were in compliance with the recommendations from the NNR2012. However, a relatively high proportion of both women and men had intakes that were higher than the maximum recommended for saturated fat and lower than recommended for fiber, and vitamin D.

## Supplementary Material

Energy and nutrient intakes in relation to National Nutrition Recommendations in a Norwegian population-based sample: the Tromsø Study 2015–16
