# Supplementary material for: Energy and nutrient intakes in relation to National Nutrition Recommendations in a Norwegian population-based sample: the Tromsø Study 2015–16
Source: Food Nutr Res. 2019 Dec 10;63:10.29219/fnr.v63.3616. doi: 10.29219/fnr.v63.3616 (PMC7007761; doi:10.29219/fnr.v63.3616)
Supplement: Energy and nutrient intakes in relation to National Nutrition Recommendations in a Norwegian population-based sample: the Tromsø Study 2015–16 [file FNR-63-3616-s001.pdf]

Supplementary table 1: Age and sex of attenders and non-attenders of Tromsø 7.

	Non-attenders	Attenders (included in final analysis)	P-value
**N**	11,508	11,425	
**Mean age (years)**	57.6 (14.1)	57.5 (10.8)	>0.5
**Mean age women (years)**	59.8 (15.2)	56.9 (10.7)	<0.001
**Mean age men (years)**	55.6 (12.7)	58.1 (11.0)	<0.001
**% women (N)**	47.5 (5465)	53.4 (6104)	<0.001

Results are given as mean (standard deviation) or as percentage (number).

Supplementary table 2: Selected characteristics of all attenders of Tromsø 7 and those included in the final study sample (after exclusion). The Tromsø Study 2015-16.

	**Attended Tromsø 7**		**P-value:**	**Completed FFQ**		**P-value:**
	**Excluded in final sample**	**Included in final sample**		**Excluded in final sample**	**Included in final sample**	
**N**	**9658**	**11,425**		**3721**	**11,425**	
**Mean age (years (SD))**	57.1 (12.1)	57.5 (10.8)	0.04	62.5 (11.9)	57.5 (10.8)	<0.001
**Women (% (N))**	51.5 (4970)	53.4 (6104)	0.004	55.3 (2056)	53.4 (6104)	0.05
**Education level (% (N))**						
- Primary	26.3 (2469)	20.6 (2327)	<0.001	33.6 (1207)	20.6 (2327)	<0.001
- Secondary	28.6 (2692)	27.1 (3064)		27.8 (997)	27.1 (3064)	
- Tertiary (short: <4 years)	17.9 (1687)	20.5 (2321)		15.6 (560)	20.6 (2322)	
- Tertiary (long: ≥4 years)	27.2 (2555)	31.8 (3590)		23.0 (825)	31.8 (3589)	
**Body mass index (mean (SD))**	27.5 (4.63)	27.2 (4.47)	<0.001	27.4 (0.08)	27.2 (4.47)	0.03
**Subjective memory complaints (% (N))**						
- Yes	44.0 (4123)	43.7 (4947)	0.71	49.0 (1775)	43.7 (4947)	<0.001
- No	56.0 (5252)	56.3 (6369)		51.0 (1845)	56.3 (6369)	
**Current smoker (% (N)):**						
- Yes	15.5 (1475)	12.6 (1429)	<0.001	12.7 (464)	12.6 (1429)	0.9

*FFQ: food frequency questionnaire; SD: standard deviation; N: number.

Supplementary table 3: Energy and nutrient intakes by sex and 10-year age groups. The Tromsø Study 2015-16.

	**N***	**Energy, MJ/d**	**Protein, E%**	**Fat, E%**	**Saturated fat, E%**	**Trans-fat, E%**	**Monounsat. fat, E%**
**RI***			**10-20**	**25-40**	**<10**	**<1**	**10-20**
**Total**	11,425	9.72 (9.67–9.78)	17.6 (17.6-17.7)	34.5 (34.3-34.6)	12.5 (12.5-12.6)	0.31 (0.30-0.31)	12.8 (12.7-12.8)
**Women**	53.4 (6104)	8.92 (8.85–8.98)	17.8 (17.7-17.9)	34.8 (34.6–34.9)	12.6 (12.6-12.7)	0.32 (0.32-0.32)	13.0 (13.0-13.0)
40-49	30.0 (1833)	9.40 (9.23-9.48)	17.4 (17.2-17.5)	35.5 (35.2-36.0)	13.0 (12.8-13.1)	0.32 (0.31-0.32)	13.4 (13.3-13.5)
50-59	29.7 (1813)	9.00 (8.88-9.13)	18.0 (17.9-18.1)	34.7 (34.5-35.0)	12.4 (12.2-12.5)	0.32 (0.31-0.32)	13.1 (13.0-13.2)
60-69	27.0 (1646)	8.63 (8.50-8.75)	18.1 (18.0-18.2)	34.6 (34.3-34.9)	12.5 (12.3-12.6)	0.32 (0.31-0.32)	12.8 (12.7-13.0)
70-79	11.1 (679)	8.35 (8.14-8.55)	18.0 (17.8-18.1)	33.6 (33.2-34.0)	12.8 (12.6-13.0)	0.33 (0.32-0.34)	11.9 (11.7-12.1)
80+	2.2 (133)	8.16 (7.69-8.64)	17.1 (16.6-17.5)	33.4 (32.5-34.4)	13.2 (12.7-13.7)	0.35 (0.32-0.37)	11.6 (11.2-11.9)
**Men**	46.6 (5321)	10.7 (10.6–10.7)	17.4 (17.3-17.4)	34.1 (33.9-34.2)	12.4 (12.3-12.5)	0.29 (0.29-0.29)	12.6 (12.5-12.7)
40-49	26.9 (1433)	11.4 (11.2-11.6)	17.0 (16.8-17.1)	34.9 (34.6-35.2)	12.8 (12.6-12.9)	0.30 (0.30-0.31)	13.1 (12.9-13.2)
50-59	27.5 (1464)	11.0 (10.8-11.1)	17.5 (17.3-17.6)	34.4 (34.1-34.6)	12.3 (12.2-12.4)	0.29 (0.28-0.30)	12.9 (12.7-13.0)
60-69	28.6 (1521)	10.3 (10.1-10.4)	17.6 (17.5-17.7)	33.6 (33.4-33.9)	12.1 (12.0-12.3)	0.29 (0.28-0.29)	12.3 (12.2-12.5)
70-79	14.4 (768)	9.62 (9.42-9.81)	17.6 (17.4-17.7)	33.0 (32.6-33.4)	12.3 (12.1-12.5)	0.29 (0.28-0.30)	11.8 (11.6-12.0)
80+	2.54 (135)	9.41 (8.95-9.86)	17.1 (16.7-17.4)	33.5 (32.7-34.4)	13.3 (12.8-13.8)	0.34 (0.31-0.36)	11.6 (11.3-12.0)

* Total is presented as number, sex- and age-specific numbers are presented as percentage (number)
* Numbers for total energy are presented as MJ/day with 95% confidence intervals.
* Numbers for macronutrients (with the exception of fiber, which is presented as g/day) are presented as energy percentage (E%) with 95% confidence intervals.
* Recommended intakes (RI) are presented as they are given in the Nordic Nutrition Recommendations 2012.

Cont. Supplementary table 3: Energy and nutrient intake by sex and 10-year age groups. The Tromsø Study 2015-16

	**Polyunsat. fat, E%**	**Omega 3, E%**	**Carbohydrates, E%**	**Fiber, g/day**	**Added sugar, E%**	**Alcohol, E%**
**RI**	**5-10**	**≥1**	**45-60**	**Women: >25<br>Men: >35**	**<10**	**<5**
**Total**	6.04 (6.01-6.07)	1.49 (1.48-1.50)	42.2 (42.1-42.3)	28.4 (28.2-28.6)	5.56 (5.50-5.63)	3.38 (3.31-3.45)
**Women**	6.01 (5.97-6.05)	1.49 (1.49-1.50)	42.0 (41.8-42.1)	28.4 (28.1-28.6)	5.45 (5.37-5.54)	2.87 (2.79-2.96)
40-49	6.00 (5.93-6.08)	1.35 (1.32-1.37)	42.3 (42.0-42.5)	28.1 (27.6-28.6)	5.96 (5.77-6.14)	2.46 (2.32-2.59)
50-59	6.09 (6.02-6.16)	1.49 (1.47-1.52)	41.4 (41.1-41.7)	29.4 (29.0-29.9)	5.16 (5.01-5.31)	3.17 (3.01-3.33)
60-69	6.09 (6.02-6.17)	1.57 (1.54-1.60)	41.4 (41.0-41.7)	28.5 (28.0-28.9)	5.08 (4.94-5.23)	3.26 (3.08-3.44)
70-79	5.69 (5.58-5.80)	1.60 (1.56-1.65)	43.4 (43.0-43.9)	26.8 (26.1-27.5)	5.65 (5.40-5.90)	2.44 (2.21-2.66)
80+	5.51 (5.28-5.75)	1.65 (1.56-1.75)	45.1 (44.1-46.1)	25.2 (23.7-26.6)	6.14 (5.55-6.73)	1.87 (1.26-2.49)
**Men**	6.07 (6.03-6.12)	1.49 (1.48-1.51)	42.4 (42.2-42.6)	28.5 (28.2-28.7)	5.68 (5.59-5.78)	3.96 (3.84-4.07)
40-49	6.09 (6.01-6.17)	1.37 (1.34-1.39)	42.8 (42.5-43.1)	28.8 (28.3-29.3)	6.06 (5.86-6.26)	3.24 (3.05-3.42)
50-59	6.15 (6.07-6.23)	1.46 (1.44-1.49)	41.9 (41.6-42.2)	29.3 (28.7-29.8)	5.36 (5.18-5.54)	4.13 (3.91-4.34)
60-69	6.13 (6.05-6.22)	1.57 (1.54-1.59)	41.9 (41.6-42.2)	28.3 (27.9-28.8)	5.40 (5.23-5.57)	4.66 (4.42-4.90)
70-79	5.86 (5.75-5.98)	1.60 (1.56-1.65)	43.4 (42.9-43.8)	27.1 (26.4-27.7)	5.90 (5.66-6.14)	3.81 (3.49-4.13)
80+	5.56 (5.29-5.84)	1.61 (1.51-1.70)	44.5 (43.6-45.4)	25.6 (23.9-27.2)	7.14 (6.47-7.80)	2.72 (2.09-3.34)

Supplementary table 4: Absolute macronutrient intakes by sex and 10-year age group. The Tromsø Study 2015-16.

	N*	Protein, g/d	Fat, g/d	Saturated fat, g/d	Trans-fat, g/d	Monounsaturated fat, g/d
**Total**	11,425	99.9 (99.3-100)	91.4 (90.7-92.0)	33.3 (33.0-33.5)	0.81 (0.80 -0.82)	33.9 (33.7 – 34.2)
**Women**	53.4 (6104)	92.9 (92.1–93.6)	84.6 (83.8-85.4)	30.8 (30.5-31.1)	0.78 (0.77-0.79)	31.6 (31.2-31.9)
40-49	30.0 (1833)	94.9 (93.5-96.3)	90.4 (88.9-91.9)	33.1 (32.5-33.7)	0.81 (0.79-0.83)	34.1 (33.5-34.7)
50-59	29.7 (1813)	94.8 (93.4-96.2)	85.2 (83.7-86.7)	30.4 (29.8-31.0)	0.78 (0.76-0.80)	32.1 (31.5-32.7)
60-69	27.0 (1646)	91.4 (90.0-92.8)	81.6 (80.0-83.1)	29.4 (28.8-30.0)	0.75 (0.73-0.76)	30.3 (29.7-30.9)
70-79	11.1 (679)	87.8 (85.5-90.0)	76.5 (74.3-78.8)	29.2 (28.2-30.1)	0.75 (0.72-0.78)	27.2 (26.4-28.1)
80+	2.2 (133)	82.6 (77.0-88.1)	74.8 (69.2-80.5)	29.6 (27.2-31.9)	0.78 (0.70-0.85)	26.0 (23.9-28.0)
**Men**	46.6 (5321)	108 (107-108)	99.1 (98.1-100)	36.1 (35.7-36.5)	0.85 (0.84 - 0.87)	36.6 (36.2-37.0)
40-49	26.9 (1433)	112 (111-114)	108 (106-110)	39.6 (38.9-40.4)	0.94 (0.92-0.96)	40.4 (39.7-41.2)
50-59	27.5 (1464)	112 (110-113)	106 (104-107)	36.9 (36.2-37.6)	0.87 (0.84-0.89)	38.5 (37.8-39.3)
60-69	28.6 (1521)	106 (104-107)	94.4 (92.6-96.1)	34.1 (33.4-34.7)	0.80 (0.78-0.83)	36 (35.2-36.7)
70-79	14.4 (768)	98.6 (96.5-101)	86.7 (84.4-89.0)	32.5 (31.5-33.5)	0 .77 (0.74-0.80)	31.0 (30.1-31.8)
80+	2.54 (135)	94.5 (89.5-99.4)	85.5 (80.7-90.4)	33.9 (31.8-36.0)	0.86 (0.78-0.93)	29.7 (27.9-31.5)

* Total is presented as number, sex- and age-specific numbers are presented as percentage (number).

Cont. supplementary table 4: Absolute macronutrient intakes by sex and 10-year age group. The Tromsø Study 2015-16.

	Polyunsaturated fat, g/d	Omega 3, g/d	Carbohydrates, g/d	Sugar, g/d	Alcohol, g/d
**Total**	16.0 (15.9 - 16.1)	3.89 (3.85-3.92)	241 (239-242)	32.9 (32.4-33.4)	11.0 (10.8-11.3)
**Women**	14.6 (14.4-14.7)	3.56 (3.52-3.60)	220 (217-221)	29.6 (29.0-30.2)	8.36 (8.12-8.60)
40-49	15.3 (15.0-15.6)	3.40 (3.32-3.47)	232 (229-236)	33.5 (32.3-34.7)	7.54 (7.13-7.95)
50-59	14.9 (14.6-15.2)	3.61 (3.54-3.69)	219 (216-223)	28.5 (27.3-29.7)	9.37 (8.89-9.84)
60-69	14.4 (14.0-14.7)	3.65 (3.57-3.73)	209 (206-212)	26.8 (25.8-27.8)	9.13 (8.63-9.64)
70-79	12.9 (12.5-13.3)	3.61 (3.48-3.73)	212 (207-218)	28.6 (27.0-30.3)	6.76 (6.15-7.37)
80+	12.3 (11.3-13.3)	3.64 (3.33-3.95)	215 (203-227)	30.0 (26.4-33.1)	4.50 (3.37-5.63)
**Men**	17.6 (17.4-17.8)	4.27 (4.21-4.32)	265 (263-267)	36.6 (35.9-37.4)	14.1 (13.7-14.5)
40-49	18.9 (18.5-19.3)	4.22 (4.12-4.31)	286 (282-291)	41.5 (39.8-43.1)	12.5 (11.7-13.3)
50-59	18.4 (18.0-18.8)	4.33 (4.23-4.43)	270 (266-274)	36.0 (34.5-37.4)	15.1 (14.3-15.9)
60-69	17.2 (16.8-17.5)	4.33 (4.23-4.42)	253 (249-257)	33.5 (32.3-34.8)	16.0 (15.2-16.8)
70-79	15.3 (14.9-15.8)	4.15 (4.02-4.28)	245 (239-250)	34.4 (32.7-36.2)	12.2 (11.2-13.2)
80+	14.2 (13.2-15.2)	4.09 (3.79-4.38)	246 (233-259)	40.6 (35.9-45.2)	8.51 (6.53-10.5)

Supplementary table 5: Absolute micronutrient intakes by sex in 10-year age group. The Tromsø Study 2015-16.

	N*	Vitamin A, RE/d	Vitamin D, µg /d	Vitamin E, α-TE/d	Vitamin B6, mg/d	Vitamin B12, µg/d	Vitamin C, mg/d	Thiamin, mg/d
**AR**		M:600<br>W:500	7.5	M:6<br>W:5	M:1.3<br>W:1.0	1.4	M:60<br>W:50	M:1.2<br>W:0.9
**RI**		M:900, W: 700	10	M:10, W: 8	M: 1.5, W: 1.2	2.0	75	M:1.3, W:1.1
**Total***	11,425	1575 (1559–1592)	14.3 (14.2-14.5)	21.2 (21.0-21.4)	2.26 (2.23-2.28)	8.53 (8.46-8.60)	156 (154-157)	2.13 (2.12-2.15)
**Women**	53.4 (6104)	1539 (1517-1560)	14.0 (13.8-14.3)	21.0 (20.7-21.3)	2.31 (2.27-2.34)	7.85 (7.76-7.93)	169 (167-172)	2.14 (2.11-2.17)
40-49	30.0 (1833)	1606 (1563-1648)	12.7 (12.3-13.2)	20.7 (20.2-21.1)	2.30 (2.24-2.36)	7.69 (7.54-7.84)	160 (156-166)	2.13 (2.08-2.18)
50-59	29.7 (1813)	1525 (1487-1564)	14.0 (13.6-14.5)	21.3 (20.8-21.8)	2.33 (2.27-2.40)	7.87 (7.71-8.03)	174 (169-179)	2.16 (2.11-2.21)
60-69	27.0 (1646)	1492 (1455-1530)	14.7 (14.2-15.2)	21.3 (20.7-21.8)	2.27 (2.21-2.34)	7.87 (7.70-8.03)	172 (167-176)	2.13 (2.07-2.18)
70-79	11.1 (679)	1501 (1436-1566)	15.5 (14.7-16.3)	20.5 (19.6-21.3)	2.32 (2.21-2.43)	8.13 (7.85-8.40)	176 (168-184)	2.16 (2.07-3.16)
80+	2.2 (133)	1570 (1445-1695)	16.7 (14.9-18.5)	21.4 (19.4-23.3)	2.38 (2.15-2.62)	8.02 (7.36-8.68)	168 (151-184)	2.18 (2.00-2.37)
**Men**	46.6 (5321)	1617 (1592-1642)	14.7 (14.4-14.9)	21.4 (21.1-21.7)	2.20 (2.17-2.22)	9.31 (9.21-9.41)	140 (138-142)	2.13 (2.10-2.15)
40-49	26.9 (1433)	1744 (1690-1799)	13.7 (13.2-14.2)	21.7 (21.1-22.2)	2.27 (2.22-2.32)	9.36 (9.17-9.56)	135 (131-139)	2.20 (2.15-2.25)
50-59	27.5 (1464)	1612 (1563-1660)	14.1 (13.6-14.6)	21.5 (20.9-22.1)	2.14 (2.09-2.18)	9.38 (9.19-9.57)	139 (135-143)	2.14 (2.09-2.18)
60-69	28.6 (1521)	1564 (1521-1606)	15.3 (14.8-15.8)	21.6 (21.0-22.2)	2.17 (2.11-2.22)	9.31 (9.12-9.50)	143 (138-147)	2.10 (2.05-2.14)
70-79	14.4 (768)	1501 (1445-1558)	15.8 (15.1-16.5)	20.6 (19.8-21.4)	2.12 (2.04-2.20)	9.16 (8.89-9.43)	143 (137-149)	2.06 (1.99-2.13)
80+	2.54 (135)	1590 (1465-1715)	17.5 (15.7-19.3)	21.4 (19.5-23.4)	1.91 (1.74-2.08)	8.90 (8.35-9.45)	142 (127-156)	1.94 (1.80-2.08)

* Total is presented as numbers, sex- and age-specific numbers are presented as percentage (number)

* Numbers are given as mg or µg per day with 95% confidence intervals.

* M: men; W: women; AR: Average requirements as they are provided by the Nordic Nutrition Recommendations 2012; RI: Recommended intakes as they are provided by the Nordic Nutrition Recommendations 2012.

Cont. Supplementary table 5: Absolute micronutrient intakes by sex and 10-year age groups. The Tromsø Study 2015-16.

	Riboflavin, mg/d	Niacin, NE/d	Folate, µg /d	Calcium, mg/d	Phosphorus, mg/d	Potassium, mg/d	Magnesium, mg/d
**AR**	M: 1.4<br>W: 1.1	M:15<br>W:12	200	500	450	None	None
**RI**	M:1.5, W:1.2	M:18, W:14	M:300, W:400/300[1]	800	600	M:3.5, W:3.1 (g/d)	M:350, W:280
**Total**	2.98 (2.96-3.01)	28.0 (27.7 - 28.2)	360 (357-363)	1155 (1146-1165)	1923 (1911-1935)	5109 (5076-5141)	431 (428-434)
**Women**	2.93 (2.89-2.97)	27.4 (27.0-27.7)	365 (361-369)	1095 (1082-1109)	1808 (1793-1823)	4864 (4821-4907)	411 (408-414)
40-49	2.85 (2.77-2.92)	27.3 (26.6-27.9)	368 (360-375)	1120 (1097-1143)	1843 (1815-1870)	4662 (4589-4736)	416 (410-422)
50-59	2.94 (2.86-3.01)	28.0 (27.4-28.7)	373 (365-380)	1095 (1071-1119)	1842 (1814-1871)	4986 (4904-5068)	423 (417-430)
60-69	2.96 (2.88-3.04)	27.1 (26.4-27.7)	358 (350-366)	1075 (1050-1100)	1779 (1750-1808)	5001 (4917-5085)	407 (400-413)
70-79	3.04 (2.91-2.16)	26.8 (25.7-27.8)	355 (343-367)	1079 (1036-1121)	1722 (1675-1770)	4829 (4696-4962)	383 (373-393)
80+	3.08 (2.81-3.34)	26.1 (23.9-28.3)	355 (329-382)	1098 (997-1199)	1656 (1540-1771)	4480 (4216-4743)	361 (339-383)
**Men**	3.05 (3.01-3.08)	28.7 (28.4-29.0)	354 (350-358)	1224 (1210-1239)	2055 (2038-2073)	5389 (5341-5438)	454 (450-458)
40-49	3.10 (3.02-3.17)	29.7 (29.0-30.3)	371 (364-379)	1294 (1265-1323)	2143 (2108-2178)	5289 (5196-5382)	469 (462-477)
50-59	3.05 (2.98-3.12)	29.6 (29.0-30.2)	360 (353-367)	1237 (1209-1264)	2116 (2083-2149)	5509 (5416-5603)	472 (465-480)
60-69	3.04 (2.96-3.11)	28.4 (27.8-28.9)	346 (339-352)	1187 (1161-1214)	2013 (1981-2045)	5466 (5373-5560)	447 (440-454)
70-79	2.99 (2.89-3.09)	26.8 (26.0-27.6)	333 (323-342)	1147 (1110-1185)	1894 (1851-1937)	5253 (5135-5370)	414 (405-423)
80+	2.87 (2.66-3.09)	23.9 (22.2-25.6)	322 (300-343)	1197 (1102-1291)	1857 (1753-1961)	5055 (4795-5314)	393 (371-414)

*1. The recommendation is 400 µg/day for premenopausal women.

*2. The recommendation is 9 mg/day for postmenopausal women.

Cont. Supplementary table 5: Absolute micronutrient intakes by sex and 10-year age groups. The Tromsø Study 2015-16.

	Iron, mg/d	Zinc, mg/d	Copper, mg/d	Selenium, µg /d
**AR**	M:7, W:10/6	M:6, W: 5	0.7	M:35, W:30
**RI**	M:9, W:15/9[2]	M:9, W:7	0.9	M:60, W:50
**Total**	12.4 (12.3–12.6)	14.4 (14.3-14.5)	1.51 (1.49-1.52)	68.1 (67.5-68.6)
**Women**	12.4 (12.2-12.7)	14.0 (13.8-14.2)	1.54 (1.52-1.57)	64.4 (63.7-65.2)
40-49	13.8 (13.2-14.4)	14.7 (14.4-15.1)	1.60 (1.56-1.65)	62.4 (61.0-63.8)
50-59	12.6 (12.2-13.0)	14.1 (13.8-14.5)	1.58 (1.53-1.62)	65.7 (64.2-67.1)
60-69	11.3 (11.0-11.7)	13.5 (13.1-13.8)	1.48 (1.44-1.53)	64.9 (63.5-66.4)
70-79	11.2 (10.6-11.8)	13.4 (12.9-14.0)	1.44 (1.37-1.52)	65.9 (63.5-68.2)
80+	11.2 (9.44-12.9)	13.0 (11.7-14.3)	1.38 (1.23-1.53)	62.0 (56.9-67.2)
**Men**	12.4 (12.2-12.6)	14.9 (14.7-15.0)	1.47 (1.45-1.49)	72.2 (71.4-73.0)
40-49	13.1 (12.8-13.4)	16.1 (15.7-16.4)	1.55 (1.51-1.60)	71.0 (69.5-72.5)
50-59	12.9 (12.5-13.3)	15.1 (14.8-15.4)	1.49 (1.46-1.53)	73.2 (71.7-74.6)
60-69	12.1 (11.8-12.5)	14.3 (14.0-14.6)	1.43 (1.39-1.47)	73.2 (71.7-74.6)
70-79	11.3 (10.8-11.8)	13.6 (13.1-14.1)	1.37 (1.31-1.43)	71.8 (69.7-73.9)
80+	9.91 (9.23-10.6)	12.6 (11.7-13.5)	1.23 (1.12-1.34)	66.0 (61.6-70.4)

Supplementary table 6: Micronutrient intakes presented in mg/µg per 10 MJ by sex and 10-year age group. The Tromsø Study 2015-16.

	N*	Vitamin A, RE/10 MJ	Vitamin D, µg/10 MJ	Vitamin E, α-TE/10 MJ	Vitamin B6, mg/d10 MJ	Vitamin B12, µg/10 MJ	Vitamin C, mg/10 MJ
**Total**	11,425	1645 (1630-1660)	15.3 (15.1-15.5)	22.2 (22.0-22.4)	2.40 (2.37-2.42)	8.82 (8.77-8.87)	166 (164-168)
**Women**	53.4 (6104)	1749 (1727-1770)	16.2 (15.9-16.5)	23.9 (23.6-24.2)	2.66 (2.62-2.70)	8.83 (8.77-8.90)	195 (192-197)
40-49	30.0 (1833)	1741 (1697-1785)	14.0 (13.5-14.5)	22.4 (21.9-22.9)	2.55 (2.47-2.63)	8.26 (8.14-8.38)	177 (173-182)
50-59	29.7 (1813)	1720 (1682-1758)	16.0 (15.5-16.6)	24.0 (23.4-24.5)	2.65 (2.58-2.72)	8.75 (8.63-8.87)	196 (192-201)
60-69	27.0 (1646)	1752 (1713-1791)	17.4 (16.8-18.0)	24.8 (24.2-25.4)	2.69 (2.61-2.77)	9.13 (9.00-9.26)	204 (198-209)
70-79	11.1 (679)	1799 (1740-1858)	19.0 (18.0-19.9)	24.7 (23.8-25.6)	2.85 (2.72-2.99)	9.71 (9.50-9.92)	212 (204-221)
80+	2.2 (133)	1957 (1834-2079)	21.2 (18.9-23.5)	26.7 (24.5-28.9)	3.00 (2.69-3.30)	9.65 (9.22-10.1)	210 (192-227)
**Men**	46.6 (5321)	1526 (1506-1546)	14.2 (13.9-14.4)	20.3 (20.1-20.6)	2.10 (2.07-2.12)	8.81 (8.74-8.88)	134 (132-136)
40-49	26.9 (1433)	1531 (1489-1573)	12.2 (11.7-12.6)	19.1 (18.7-19.5)	2.03 (1.98-2.07)	8.26 (8.14-8.39)	121 (117-124)
50-59	27.5 (1464)	1471 (1434-1508)	13.1 (12.7-13.6)	19.7 (19.3-20.2)	2.05 (2.02-2.09)	8.60 (8.47-8.73)	129 (125-132)
60-69	28.6 (1521)	1540 (1502-1577)	15.3 (14.8-15.8)	21.3 (20.7-21.8)	2.14 (2.09-2.20)	9.10 (8.96-9.23)	141 (137-145)
70-79	14.4 (768)	1566 (1515-1617)	16.7 (15.9-17.4)	21.6 (20.8-22.3)	2.23 (2.16-2.31)	9.55 (9.35-9.75)	150 (145-156)
80+	2.54 (135)	1685 (1578-1792)	18.9 (16.9-20.8)	22.8 (20.9-24.8)	2.05 (1.87-2.22)	9.46 (9.10-9.82)	150 (136-165)

* Total is presented as numbers, sex- and age-specific numbers are presented as percentage (number)

*Numbers are given as mg or µg per day with 95% confidence intervals

Cont. supplementary table 6: Micronutrient intake presented in mg/µg per 10 MJ by sex and 10-year age group. The Tromsø Study 2015-16.

	Thiamin, mg/10 MJ	Riboflavin, mg/10 MJ	Niacin, NE/10 MJ	Folate, µg /10 MJ	Calcium, mg/10 MJ	Phosphorus, mg/10 MJ	Potassium, mg/10 MJ	Magnesium, mg/10 MJ
**Total**	2.26 (2.24-2.28)	3.14 (3.11-3.17)	29.7 (29.4-29.9)	379 (376-382)	1186 (1179-1193)	1990 (1984-1995)	5355 (5330-5379)	448 (447-449)
**Women**	2.47 (2.43-2.50)	3.35 (3.31-3.40)	31.6 (31.2-32.0)	417 (413-421)	1217 (1208-1227)	2033 (2025-2041)	5537 (5504-5571)	464 (462-466)
40-49	2.35 (2.29-2.42)	3.12 (3.04-3.21)	30.2 (29.4-31.0)	403 (395-411)	1192 (1174-1210)	1976 (1962-1990)	5053 (4996-5109)	449 (446-453)
50-59	2.45 (2.40-2.51)	3.32 (3.24-3.39)	31.9 (31.2-32.5)	419 (413-426)	1205 (1188-1222)	2051 (2037-2065)	5604 (5543-5665)	473 (470-476)
60-69	2.52 (2.46-2.58)	3.47 (3.39-3.56)	32.8 (31.5-32.9)	421 (413-429)	1231 (1212-1249)	2066 (2051-2080)	5875 (5807-5943)	474 (470-478)
70-79	2.65 (2.54-2.75)	3.69 (3.55-3.83)	33.0 (31.8-34.3)	431 (419-444)	1270 (1240-1299)	2063 (2041-2085)	5848 (5752-5944)	462 (456-468)
80+	2.74 (2.51-2.97)	3.81 (3.51-4.11)	33.0 (30.1-35.8)	442 (415-470)	1300 (1235-1364)	2007 (1954-2060)	5546 (5374-5718)	444 (432-456)
**Men**	2.03 (2.01-2.05)	2.90 (2.87-2.94)	27.5 (27.2-27.7)	336 (333-339)	1149 (1139-1160)	1940 (1932-1949)	5145 (5110-5180)	430 (428-431)
40-49	1.95 (1.92-1.99)	2.75 (2.69-2.81)	26.5 (26.0-26.9)	329 (324-334)	1136 (1118-1155)	1891 (1876-1905)	4702 (4643-4760)	415 (412-419)
50-59	1.97 (1.94-2.00)	2.82 (2.76-2.87)	27.4 (26.9-27.8)	331 (327-336)	1131 (1111-1151)	1942 (1925-1958)	5102 (5036-5169)	434 (430-437)
60-69	2.08 (2.03-2.12)	3.00 (2.93-3.07)	28.2 (27.6-28.7)	340 (335-346)	1151 (1132-1170)	1965 (1949-1981)	5381 (5313-5449)	438 (434-441)
70-79	2.17 (2.11-2.23)	3.13 (3.04-3.22)	28.4 (27.7-29.2)	349 (341-356)	1185 (1157-1212)	1976 (1954-1998)	5539 (5452-5626)	434 (429-439)
80+	2.08 (1.95-2.21)	3.09 (2.89-3.29)	25.8 (24.1-27.4)	342 (325-359)	1263 (1195-1331)	1971 (1923-2020)	5417 (5259-5575)	417 (407-427)

Cont. Supplementary table 6: Micronutrient intake presented in mg/µg per 10 MJ by sex and 10-year age group. The Tromsø Study 2015-16.

	Iron, mg/10 MJ	Zinc, mg/10 MJ	Copper, mg/10 MJ	Selenium, µg/10 MJ
**Total**	13.1 (12.9-13.3)	15.1 (15.0-15.3)	1.59 (1.57-1.61)	71.5 (71.0-72.0)
**Women**	14.2 (14.0-14.6)	16.0 (15.8-16.3)	1.77 (1.74-1.80)	73.7 (72.9-74.6)
40-49	15.1 (14.4-15.9)	16.2 (15.8-16.7)	1.77 (1.71-1.83)	68.3 (66.6-69.9)
50-59	14.3 (13.8-14.8)	15.9 (15.5-16.3)	1.78 (1.73-1.83)	73.9 (72.5-75.3)
60-69	13.4 (13.0-13.8)	15.8 (15.4-16.2)	1.75 (1.70-1.81)	76.7 (75.1-78.2)
70-79	13.7 (12.9-14.4)	16.4 (15.7-17.1)	1.76 (1.67-1.85)	80.4 (77.8-82.9)
80+	14.3 (11.7-16.9)	16.1 (14.5-17.7)	1.74 (1.53-1.96)	77.1 (71.4-82.8)
**Men**	11.8 (11.7-12.0)	14.1 (13.9-14.2)	1.39 (1.37-1.41)	68.9 (68.3-69.6)
40-49	11.6 (11.3-11.9)	14.3 (14.0-14.5)	1.38 (1.35-1.42)	63.2 (62.0-64.3)
50-59	12.0 (11.6-12.3)	13.9 (13.7-14.1)	1.38 (1.35-1.41)	67.5 (66.5-68.6)
60-69	12.0 (11.6-12.4)	14.0 (13.7-14.3)	1.41 (1.37-1.44)	72.3 (71.0-73.5)
70-79	11.9 (11.4-12.4)	14.2 (13.8-14.7)	1.44 (1.38-1.49)	75.7 (73.9-77.5)
80+	10.5 (9.98-11.1)	13.4 (12.7-14.2)	1.30 (1.20-1.40)	70.1 (66.8-73.5)

Supplementary figure 1:

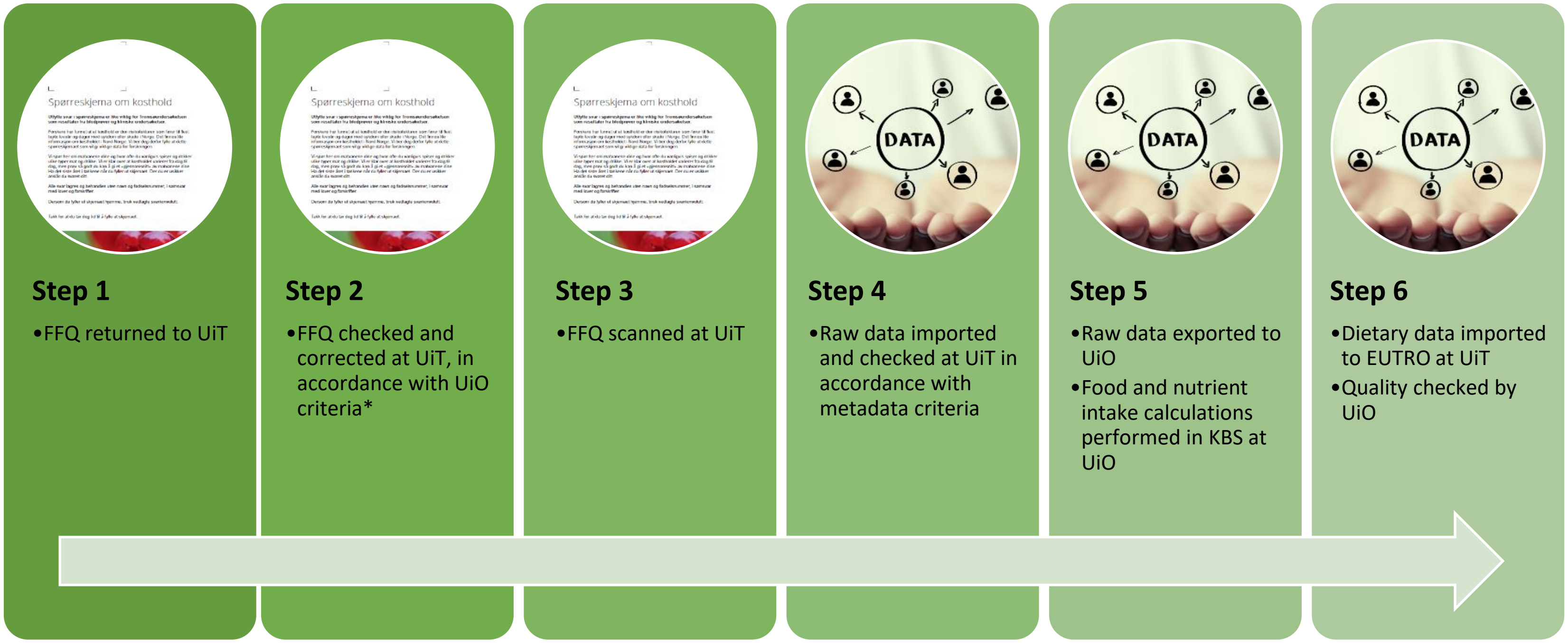

Correction guidelines (food frequency questionnaire) from the University of Oslo:

- If two frequency fields were marked, one clearly marked and one scribbled out, choose the one clearly marked.
- If two frequency fields were marked, and both are clearly marked, do the following:
    - If the two marked fields are adjacent, choose the one with the lowest value
    - If one of the two marked fields was "seldom/never" or zero, and there were no marks on "amount", choose seldom/never
    - If one of the two marked fields was "seldom/never" or zero, and there was a mark on "amount", choose the marked field with a frequency value other than "seldom/never" or zero.
- Be sure to check specifically that unclear and skewed markings were scanned and registered properly.
- Information from the open field questions was manually included in the database.
